# Preventing Zoonotic Influenza Virus Infection

**DOI:** 10.3201/eid1206.051576

**Published:** 2006-06

**Authors:** Alejandro Ramirez, Ana W. Capuano, Debbie A. Wellman, Kelly A. Lesher, Sharon F. Setterquist, Gregory C. Gray

**Affiliations:** * University of Iowa College of Public Health, Iowa City, Iowa, USA;; † Iowa State University College of Veterinary Medicine, Ames, Iowa, USA

**Keywords:** zoonoses, influenza viruses, swine, occupational exposure, communicable diseases, emerging, agriculture, seroepidemiologic studies, veterinarians, gloves, masks, dispatch

## Abstract

We evaluated 49 swine industry workers and 79 nonexposed controls for antibodies to swine influenza viruses. Multivariate modeling showed that workers who seldom used gloves (odds ratio [OR] 30.3) or who smoked (OR 18.7) most frequently had evidence of previous H1N1 swine virus. These findings may be valuable in planning for pandemic influenza.

In the United States, influenza viruses are estimated to cause 36,000 human deaths and 200,000 hospitalizations annually (*1**–**5*). The current outbreaks of avian influenza in Asia and Eastern Europe remind us of the zoonotic potential of these viruses. Swine cells express sialic acids that can be receptors for swine, human, and avian influenza strains and facilitate cross-species influenza transmission and the genesis of novel influenza strains. Reported cases of human-to-swine and swine-to-human influenza transmission illustrate this potential (*6**,**7*).

Persons who work in enclosed livestock buildings (confinement workers) have among the highest risk of becoming infected with swine influenza virus. Their work involves close contact with many swine, including sick ones. The purpose of this cross-sectional study was to learn if these workers had evidence of previous swine influenza virus infection and, if so, to determine factors that cause them to be at increased risk.

## The Study

Iowa is the top swine-producing state in the United States and markets ≈25 million swine a year. From November 2004 to March 2005, we recruited confinement workers. Site selection was based on the authors' community contacts and opportunities to invite workers to participate. Local veterinary clinics advertised the study and permitted enrollment at their facilities. This study was approved by the University of Iowa's institutional review board.

Persons were eligible to participate in the study if they had worked in a swine confinement facility in the past 12 months. Participants completed a questionnaire and permitted blood sample collection on enrollment. The questionnaire captured demographic, medical, and occupational data including influenza immunization history, swine occupational exposures, and use of protective equipment (gloves and masks). Nonexposed controls were enrolled during a concurrent study of University of Iowa faculty, staff, and students (*8*).

Serum samples were studied by using a hemagglutination inhibition (HI) assay against 2 recently circulating swine strains, A/Swine/WI/238/97 (H1N1) and A/Swine/WI/R33F/01 (H1N2), and 1 human influenza virus strain, A/New Caledonia/20/99 (H1N1). The swine H1N1 strain represents a lineage of virus that has been circulating among US swine for 70 years. The swine H1N2 strain first appeared in US swine in 1999. HI titer results are reported as the reciprocal of the highest dilution of serum that inhibited virus-induced hemagglutination of a 0.65% solution of guinea pig erythrocytes for human influenza and 0.5% solution of turkey erythrocytes for swine influenza.

Specimen laboratory results were examined for their statistical association with demographic, immunization, occupational, and other behavioral risk factors. Confinement workers were queried about the nature of their work and whether they had used protective equipment. Because incidence of high titers was low or nonexistent in most groups, H1N1 titers >10 were grouped. The resulting categories were <10, 10, and >10. Wilcoxon rank sum and χ^2^ statistic or 2-sided Fisher exact test were used to access bivariate risk factor associations. Depending on the nature of the data and modeling assumptions, proportional odds modeling or logistic regression was used to adjust for multiple risk factors. Final multivariate models were designed by using a saturated model and manual backwards elimination. Analyses were performed by using SAS version 9.1 (SAS Institute, Inc., Cary, NC, USA). Questionnaires were made available in both English and Spanish. Site selections were based on personal contacts in 3 completely different areas.

Forty-nine confinement workers and 79 nonexposed controls were enrolled in the study. The distribution of ages was similar for the 2 groups, but the confinement workers were more likely to be male and Hispanic and less likely to have received influenza vaccination (Table 1).

**Table 1:** Characteristics of swine workers and controls, Iowa, 2004–2005

Variable	Total	Study sample, n (%)	
		Swine workers (n = 49)	Controls (n = 79)*
Age group (y)			
	<29	46	15 (30.6)	31 (39.2)
	29–42	40	12 (24.5)	28 (35.4)
	>42	42	22 (44.9)	20 (25.3)
	Mean age (y)		40.3	35.3
Sex†			
	Male	63	37 (75.5)	26 (32.9)
	Female	65	12 (24.5)	53 (67.1)
Race†			
	American Indian/Alaska Native	1	0	1 (1.3)
	Asian	2	1 (2)	1 (1.3)
	African American	18	0	18 (22.8)
	Native Hawaiian/Other Pacific Islander	2	0	2 (2.5)
	White	105	48 (98)	57 (72.2)
Ethnicity†			
	Hispanic/Latino	12	10 (21.7)	2 (2.5)
	Non-Hispanic/Latino	113	36 (78.3)	77 (97.5)
Served in military			
	Yes	12	5 (10.4)	7 (8.9)
	No	115	43 (89.6)	72 (91.1)
Take medications that weaken immune system			
	Yes	3	2 (4.2)	1 (1.3)
	No	122	44 (91.7)	78 (98.7)
	Do not know	2	2 (4.2)	0 (0)
Have heart or vascular disease			
	Yes	3	2 (4.2)	1 (1.3)
	No	124	46 (95.8)	78 (98.7)
Have any chronic lung problems such as asthma or emphysema			
	Yes	5	2 (4.2)	3 (3.8)
	No	122	46 (95.8)	76 (96.2)
Received the 2001–02 influenza vaccine			
	Yes	37	6 (12.5)	31 (39.2)
	No	90	42 (87.5)	48 (60.8)
Received the 2002–03 influenza vaccine†			
	Yes	36	5 (10.4)	31 (39.2)
	No	91	43 (89.6)	48 (60.8)
Received the 2003–04 influenza vaccine†			
	Yes	43	8 (16.7)	35 (44.3)
	No	84	40 (83.3)	44 (55.7)
Received the 1976–77 influenza vaccine†			
	Yes	2	1 (2.1)	1 (1.3)
	No	119	41 (85.4)	78 (98.7)
	Do not know	6	6 (12.5)	0
Smoked >5 packs of cigarettes in past year†			
	Yes	14	9 (18.4)	5 (6.3)
	No	114	40 (81.6)	74 (93.7)

*Not exposed to swine. †Significantly different than controls at α = 0.05.

Swine confinement workers were categorized by type of work, frequency of contact with swine, use of gloves, and use of masks. The question "When working with sick or diseased swine, how often do you wear gloves?" explained the most variation in swine H1N1 antibody titers and was included in the best fit model. Workers who sometimes or never used gloves were significantly more likely (odds ratio [OR] 30.3, 95% confidence interval [CI] 3.8–243.5) to have elevated titers than the nonexposed controls (Table 2). These workers also were significantly more likely (OR 12.7, 95% CI 1.1–151.1) (data not shown) to have elevated titers than the other confinement workers who used gloves most of the time or always. Workers who reported smoking also had high OR (data not shown) for elevated titers.

**Table 2:** Odds ratios for increased serologic response against swine H1N1 influenza virus by hemagglutination inhibition assay

Variable	n	Swine H1N1*			
		Titer >10, n (%)	Titer >20, n (%)	Bivariate OR (95% CI)	Multivariate OR (95% CI)
Age group (y)					
	<29	40	3 (7.5)	1 (2.5)	1.2 (0.2–6.1)	3.5 (0.4–30.6)
	29–42	46	3 (6.5)	1 (2.2)	Reference	Reference
	>42	42	9 (22)	6 (14.6)	4.2 (1.1–16.8)†	6.1 (0.9–41.3)
Sex					
	Male	63	13 (21)	7 (11.3)	8.4 (1.8–38.7)†	7 (0.9–52.1)
	Female	65	2 (3.1)	1 (1.5)	Reference	Reference
Swine exposure					
	Swine workers occasionally or never use gloves	34	12 (35.3)	7 (20.6)	21 (4.4–100.8)†	30.3 (3.8–243.5)†
	Swine workers usually or always use gloves	14	1 (7.1)	0	2.8 (0.2–34.2)	2.4 (0.1–40.9)
	Controls not exposed to swine	79	2 (2.6)	1 (1.3)	Reference	Reference
Smoked >5 packs of cigarettes in past year					
	Yes	14	4 (28.6)	3 (21.4)	4 (1.1–14.5)†	18.7 (2.5–141.3)†
	No	114	11 (9.7)	5 (4.4)	Reference	Reference
Received 2002–03 influenza vaccine					
	Yes	36	4 (11.4)	1 (2.9)	1 (0.3–3.4)	–
	No	91	10 (11)	7 (7.7)	Reference	–
Received 2003–04 influenza vaccine					
	Yes	43	6 (14.3)	3 (7.1)	1.6 (0.5–4.8)	16.3 (2.5–107.4)†
	No	84	8 (9.5)	5 (6)	Reference	Reference
Elevated titer human H1N1 (>40)					
	Positive	39	2 (5.3)	1 (2.6)	0.3 (0.1–1.5)	–
	Negative	89	13 (14.6)	7 (7.9)	Reference	–

*OR, odds ratio; CI, confidence interval; by using proportional odds model, these titers were grouped: <10, 10, >10. †Significant odds for increased serologic response, p<0.05.

Multivariate analysis also showed that persons who had received the 2003–04 influenza vaccine were significantly more likely to have elevated titers (>10) against swine H1N1 virus (Table 2) as well as swine H1N2 (data not shown). Although cross-reaction with 1 of the viruses in the 2003–04 vaccine or a circulating influenza virus may explain this occurrence, higher titers would have been expected for all vaccinated persons (including controls), but such higher titers were not observed (Figure). We suggest that this result represents other behavior or health-related confounders not identified in the questionnaire for this study.

**Figure:**
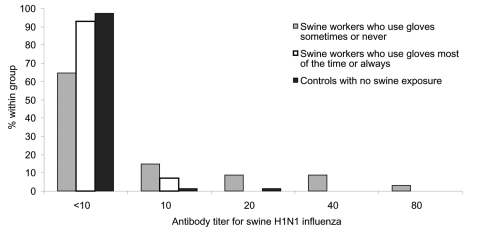
Variation in serologic response against swine H1N1 influenza virus and frequency of glove use by swine workers.

## Conclusions

These data suggest, like previous studies (*8**–**10*), that swine confinement workers are at increased risk for zoonotic influenza infection. However, our data are among the first to evaluate swine confinement workers, our sample size was small (not likely representative of all swine workers), and exposure data were self-reported. Confinement workers, in contrast to other swine occupations, are difficult to reach because of language barriers, on-farm policies regarding visitors (biosecurity protocols), and lack of trust in the public health sector.

Several studies have documented smoking as a risk factor for human influenza virus infection (*11**–**13*). However, we believe our data are the first evidence that smoking also increases the risk for swine influenza virus infections. We believe that this increased risk may be because the workers' oral mucosa are exposed to swine influenza virus after handling pigs.

This study's chief unique contribution is the evidence that use of gloves during swine confinement work noticeably decreases the risk for swine influenza virus infection. Thus, a simple personal protective measure might do much to reduce swine-to-human virus transmission. Future larger studies of swine confinement workers are needed to validate our findings and to better quantify risk factors for this population.

Individual behavior strongly influences influenza virus transmission (*5*). The national strategy for pandemic influenza highlights worker education and emphasizes individual responsibilities in preventing the spread of infection (*14*). Should a virulent, novel zoonotic influenza virus enter swine confinement facilities and spread among concentrated swine populations, the impact would be grave. Surveillance for zoonotic influenza virus therefore must be routinely conducted among agricultural workers. Also, use of personal protective equipment, frequent hand washing, and restrictions on smoking in or around swine facilities should be encouraged. Further, such workers should be included in state and federal pandemic plans as a high-risk group designated to receive annual influenza vaccines and antiviral drugs during pandemics.
